# Effects of an anthropogenic diet on indicators of physiological challenge and immunity of white ibis nestlings raised in captivity

**DOI:** 10.1002/ece3.6548

**Published:** 2020-07-14

**Authors:** Caroline R. Cummings, Sonia M. Hernandez, Maureen Murray, Taylor Ellison, Henry C. Adams, Robert E. Cooper, Shannon Curry, Kristen J. Navara

**Affiliations:** ^1^ Department of Poultry Science The University of Georgia Athens GA USA; ^2^ Warnell School of Forestry and Natural Resources The University of Georgia Athens GA USA

**Keywords:** bactericidal assay, corticosterone, phytohemagglutinin, urbanization

## Abstract

When wildlife forage and/or live in urban habitats, they often experience a shift in resource availability and dietary quality. Some species even use human handouts, such as bread, as well as human refuse, as a large part of their new diets; yet the influences of this nutritional shift on health and survival remain unclear. American white ibises are increasingly being seen in urban areas in Florida; they collect handouts, such as bread and other food items, from humans in parks, and are also found foraging on anthropogenic sources in trash heaps. We hypothesized that the consumption of these new anthropogenic food sources may trigger increases in indicators of physiological challenge and dampen immune responses. We tested this experimentally by raising 20 white ibis nestlings in captivity, and exposing 10 to a simulated anthropogenic diet (including the addition of white bread and a reduction in seafood content) while maintaining 10 on a diet similar to what ibises consume in more natural environments. We then tested two indicators of physiological challenge (corticosterone and heat shock protein 70), assessed innate immunity in these birds via bactericidal assays and an in vitro carbon clearance assay, and adaptive immunity using a phytohemagglutinin skin test. The anthropogenic diet depressed the development of the ability to kill *Salmonella paratyphi* in culture. Our results suggest that consuming an anthropogenic diet may be detrimental in terms of the ability to battle a pathogenic bacterial species, but there was little effect on indicators of physiological challenge and other immunological measures.

## Graphical abstract

White ibises are increasingly found foraging in urban environments, and previous work has shown that ibises in urban environments have elevated bactericidal capacities and depressed stress responses. We tested whether intake of an anthropogenic diet may explain those differences. An anthropogenic diet inhibited the development of bactericidal capacity against a pathogenic strain of bacteria.

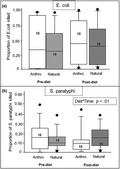

## 1 INTRODUCTION

Urban habitats present many changes for wildlife, including a change in the abundance and makeup of resources provided both intentionally (e.g., bread to birds in parks) and unintentionally (e.g., waste from trash cans) to wildlife. As a result of resource provisioning, many species experience a shift in the types of foods they consume. For example, urban common ravens consumed more trash than natural counterparts, and urban red foxes consumed more scavenged meat, compost, pet food, and berries than peri‐urban foxes (Contesse, Hegglin, Gloor, Bontadina, & Deplazes, 2004; Kristan, Boarman, & Crayon, 2004). It is unclear whether the consumption of these anthropogenic food sources is beneficial or detrimental to wildlife. On the one hand, consistent and predictable access to anthropogenic foods has the potential to alleviate the effects caused by lack of natural food and nutrient availability, often the most limiting factor for wildlife (Ostfeld & Keesing, 2000; Sinclair & Krebs, 2002). Sufficient resource abundance may allow allocation of more energy towards energetically demanding processes such as immune function, self‐care, and reproduction, and may ultimately improve overall condition (Houston, McNamara, Barta, & Klasing, 2007; Houston, McNamara, & Hutchinson, 1993; Nilsson, 2002). For example, access to anthropogenic resources led to earlier breeding in Florida scrub jays (Schoech, Bowman, & Reynolds, 2004), increased fledging success for common ravens, and banded mongooses with access to supplemental resources carried more fetuses and had higher body condition scores (Otali & Gilchrist, 2004; Webb, Boarman, & Rotenberry, 2004). Appropriate and abundant resources may enhance immune function, as investment in immune function becomes less costly with improved condition and greater access to energy (Møller et al., 1998).

However, there are also potential negative impacts of constantly consuming anthropogenic diets. Consuming these diets can trigger changes in hormones and other physiological mediators because animals frequently interact with humans and other wildlife while consuming those resources. Additionally, urban diets may often lack essential nutrients that are typically provided through natural diets, such as protein (Murray et al., 2015). Consuming a suboptimal diet can have detrimental effects on wildlife health, particularly on immunity, as many aspects of immune function require specific nutrients (e.g., T cells require specific protein levels to properly function) (Cooper, Good, & Mariani, 1974). Additionally, energetically insufficient diets can lead to an immune‐deficient animal, particularly when investment of available energy is allocated towards other processes, such as growth, reproduction, and ornamentation (Forbes et al., 2016; Lochmiller & Deerenberg, 2000; Taylor et al., 2013).

Relationships between diet and immunity have been studied in several wild species. For example, bobwhite quail consuming diets lower in protein had depressed lymphoid function (Lochmiller, Vestey, & Boren, 1993); urban coyotes consuming greater proportions of urban resources had decreased body condition and greater prevalence of disease, and protein content has been hypothesized as the main factor driving this relationship (Murray, Hill, Whyte, & Clair, 2016). Additionally, changes in micronutrient levels in the diet can have implications on wildlife physiology. For example, harp seals fed freshwater smelt and herring, which naturally contains thiaminase, experienced plasma electrolyte imbalances, central nervous system effects, and even death due to thiamin deficiency (Geraci, 1972). Moreover, female lesser black‐backed gull supplemented with carotenoid‐rich food had reduced immunoglobulin levels than nonsupplemented birds, indicating suppressed immunity (Blount et al., 2002). So, if consuming anthropogenic food sources results in deficiencies in key nutrients, animals consuming these anthropogenic resources may suffer reductions in body condition, overall health, and ability to combat pathogens (Becker, Streicker, & Altizer, 2015).

American white ibises, *Eudocimus albus*, are wading birds commonly found in wetlands along the coast of the southeastern United States. While they continue to breed in mixed‐flock rookeries in freshwater wetlands, white ibises have recently become prevalent foragers during the day in urban habitats throughout South Florida (Boyle, Dorn, & Cook, 2014). Florida is one of the most rapidly urbanizing landscapes in the United States, and changes to the landscape have caused wetland degradation driven by anthropogenic factors such as development, contamination, and draining (Chimney & Goforth, 2001; Dorn et al., 2011). In response to wetland degradation, ibis breeding numbers have declined significantly over the past 80 years in the state of Florida, and they are now listed as a Species of Concern (Crozier & Gawlik, 2003; Frederick, Gawlik, Ogden, Cook, & Lusk, 2009). White ibis naturally prey on aquatic invertebrates, fish, and insects by probing the substrate with their long, decurved bill. Successful utilization of this tactilely driven foraging mechanism is dependent on specific water levels and soft soil to find prey, which means ibises display nomadic behaviors to search for appropriate foraging areas throughout the year (Bancroft, Gawlik, & Rutchey, 2002; Kushlan, 1986). As a result of recent changes to wetland ecosystems, water levels are often suboptimal for natural foraging and natural prey abundance for white ibises fluctuates more than in the past (Dorn et al., 2011).

Ibises are now commonly found foraging throughout urban parks, zoos, and residential neighborhoods, where the bulk of their diet comes directly from human handouts (e.g., park goers tossing bread) and neighborhoods or landfills where they forage on anthropogenic food and/or waste (Murray et al., 2018). They maintain their natural foraging behavior and consume terrestrial invertebrates and aquatic organisms living in urban water sources; however, some flocks appear to be dependent solely on human handouts and supplemental resources (Welch, 2016). Variation in the isotopic signatures of ibis diets (i.e., δ^13^C and δ^15^N) increases as the level of the urbanization of the capture site increases, reflecting changes in ibis diet as habitat changes (Curry, 2017). Additionally, ibises captured from highly urbanized sites (as determined by surrounding land cover) assimilated more anthropogenic resources and less dietary protein (δ^15^N) (Murray et al., 2018).

How ibis health may change in response to anthropogenic resources and synurbanization has been studied in concurrent field studies, and the effects of this shift in habitat and diet composition vary. Ibises that consumed more anthropogenic food had lower body conditions (Murray et al., 2018), and birds in urban environments had higher prevalence of *Salmonella* infection (Hernandez et al., 2016). However, birds consuming more anthropogenic diets also had *lower* ectoparasite scores. Thus, it is still unclear whether anthropogenic diets are beneficial or detrimental to health of white ibises, and there is a need to examine how these diets influence physiological indicators of health and immunity.

One of the biggest challenges in assessing urban diets and their effects on urban wildlife health is the naturally confounding factors associated with field settings. While urban resources have been shown to both improve and impair fitness, the cause of these effects cannot be definitively assigned, as field or observational studies do not typically allow for isolation and manipulation of just one variable of interest. Additionally, wildlife nutritional studies often require repeated field observations and the best physiological assessments of nutrition require invasive sampling techniques, making robust results hard to obtain (Murray, Becker, Hall, & Hernandez, 2016; Page & Underwood, 2006). To isolate the main effect of diet quality on ibis health, we raised a captive colony of ibis nestlings and subjected half of the nestlings to an anthropogenic diet designed to mimic urban resources in the wild.

We measured the effects of diet on four different measure of immune function to best capture the effects of diet on the diverse arms of the immune system, which may not all respond identically. Because resource provisioning can affect glucocorticoid levels, and because the immune system and the hypothalamic–pituitary–adrenal (HPA) axis are tightly connected, we also quantified corticosterone concentrations, as corticosterone has the potential to both enhance and suppress particular immune components (Demas, Zysling, Beechler, Muehlenbein, & French, 2011). For example, elevated corticosterone levels have been shown to both diminish (Gao, Sanchez, & Deviche, 2017; Matson, Tieleman, & Klasing, 2006) and enhance innate immune function (Merrill, Levinson, O'Loghlen, Wingfield, & Rothstein, 2014) measured by bacterial killing assays. We analyzed heat shock protein levels, which are released in response to physiological challenges that can provoke cellular damage—including nutritional deficits—at a slower rate than corticosterone (see Herring and Gawlik (2007) for a review of the use of stress proteins in avian ecology) (Moreno, Merino, MartÍnez, Sanz, & Arriero, 2002). We hypothesized that consumption of an experimental anthropogenic diet would significantly influence corticosterone and HSP‐70 concentrations, as well as immune functions in white ibises.

## 2 METHODS

### 2.1 Nestling husbandry

In April 2017, we collected 20 free‐ranging American white ibis nestlings (ranging from 10 to 14 days of age) from the 6th Bridge rookery, in Broward County, FL. Upon collection, nestlings were color banded for individual identification and transported to Athens, Georgia, where they were maintained at the Poultry Diagnostic and Research Center (PDRC) at the University of Georgia from April 2017–October 2017. At the PDRC, all birds were initially housed inside a modified chicken house, in a single pen (*H* × *W* × *L* = 3.9 × 3.9 × 5.9 m). The cement floor was covered with artificial grass matting, and the birds were provided with full‐spectrum lights (ZooMed Avian Sun 5.0), perches of multiple sizes and heights, a wading pool, constant clean drinking water in separate bowls, natural and synthetic browse to promote nestling development and provide space for hiding. Initially, the room temperature was tightly controlled with heaters to accommodate a nestling's inability to thermoregulate. At admission to PDRC, all birds were fed a piscivore gruel (Emeraid) by gavage tube. After 3 days, they were fed by syringe, and by day 7, most were transitioned to hand‐feeding (Table 1). At captive day 21, the birds were divided into two groups housed in identical pens by age, such that larger/older birds that were capable of feeding independently off of platters were grouped together and those still requiring syringe feeding formed a second group. These pens were identical in size to the pretreatment pens. We determined the sexes of all birds using standard molecular techniques (Fridolfsson & Ellegren, 1999).

**TABLE 1:** A description of the dietary contents and timing as the ibis developed, up until the point that they were split into two dietary treatment groups (anthropogenic and natural diet)

Diet phase	*N*	CaptiveDays	Delivery	Components
Gruel (*n* = 20)	20	0–14	Oral gavage or hand fed	Lake smelt, mazuri flamingo breeder pellets, dried egg yolk, CaCO3, Vionate, Stuart Thiamin E paste, Solgar chelated copper, water
Solid diet (*n* = 20)	20	15–45	Platters	Lake smelt, mazuri flamingo breeder pellets, dried egg yolk, CaCO3, Vionate, Stuart Thiamin E paste, Solgar chelated copper, water
Solid diet + seafood (*n* = 20)	20	46–113	Platters	Lake smelt, shrimp, whole crayfish, mazuri flamingo breeder pellets, fresh egg yolk, vionate
Final natural diet (*n* = 10)	10	113–171	Platters	20% lake smelt, 20% shrimp, 20% whole crayfish, 20% mazuri flamingo breeder pellets, 20% fresh egg yolk, <1% vionate
Final anthropogenic diet (*n* = 10)	10	113–171	Platters	6.6% lake smelt, 6.6% shrimp, 6.6% whole crayfish, 20% mazuri flamingo breeder pellets, 20% fresh egg yolk, <1% vionate, 40% white bread

By captive day 93, all birds were feeding exclusively from platters and were randomly assigned to one of two pens to prevent size/age bias while ensuring equal sex ratios within each pen to prevent sex bias (five females, five males; *n* = 10 per pen). These pens would eventually represent the diet treatments the birds received. As the seasons changed, birds were maintained at temperatures representative of ibis natural habitat, ranging from 24 to 30°C, using heaters, fans, and cooling towers as necessary and constant monitoring of ambient temperature and humidity with environmental thermometers. Each pen was divided into two halves with a plastic curtain, and the birds were gently shuttled to one half while the other was cleaned daily. Artificial grass matting, water bowls, wading pools, browse, and any other enrichment items were taken outside the building and cleaned thoroughly with soap and water, sprayed with 10% bleach solution, rinsed, and fully dried (Fowler & Miller, 2012). The cleaning schedule rotated so that all parts of the pen were cleaned at least every 3 days. Once cleaned, materials were put back in the pen and natural browse was replaced. Because American white ibis develop rapidly, the husbandry, enrichment methods, and diets were constantly adjusted to meet their needs (Fowler & Miller, 2012).

### 2.2 Diet

The diets for both treatment groups were based on the diets of ibis raised in captivity at zoological facilities, but were modified to meet nutritional requirements (Table 2). In general, once birds were self‐feeding from platters, they were consuming a diet composed of nutritionally balanced commercially available pellets designed for growing flamingos (Mazuri Flamingo Breeder) to which the following were added: seafood (shrimp and smelt), cooked egg (for additional protein), water, and vitamin/micronutrient supplements. Until 113 days of captivity, all birds were maintained on a balanced diet representative of components and nutrients the birds would be consuming in their natural habitats. After this point, one pen was randomly assigned to the anthropogenic diet treatment and gradually received a greater proportion of simple carbohydrates (white bread) while the protein content was simultaneously decreased by reducing the amount of seafood. By 17 weeks of captivity (captive day 120), the nestlings fed the anthropogenic diet were consuming a diet consisting of 40% white bread by weight. This diet was designed to mimic diets wild ibis consume in urban habitats, which are typically lower in protein and higher in carbohydrates due to the increase in human handouts and consumption of refuse as opposed to natural foraging of aquatic invertebrates. A summary of the diets can be found in Table 1, and the nutritional content of the experimental diets is listed in Table 2. We measured body condition of the birds before and during the diet to both test the effects of the diet and ensure the health of the birds.

**TABLE 2:** A summary of the diet composition for the white ibis nestlings and the delivery methods used throughout the experimental design

	Natural Diet	Anthropogenic Diet
Dry Matter, %	37.68	38.07
Crude Protein, %	38.46	20.32
Crude Fat, %	8.23	15.73
Carbohydrates, %	15.33	38.43
Crude Fiber, %	1.72	1.05
Ca, %	3.07	1.27
P, %	1.23	0.64
Mg, %	0.2	0.13
K, %	0.75	0.8
Na, %	0.75	0.59
Fe, mg/kg	393	180
Zn, mg/kg	91	46
Cu, mg/kg	20	8.6
Mn, mg/kg	115	53
Se, mg/kg	0.89	0.32
Vitamin A, IU/g	26	14.6
Thiamin (B1), mg/kg	11.4	9.18
Vitamin C, mg/kg	6.62	104.18
Vitamin D3, IU/g	4.04	2.46
Vitamin E, mg/kg	87.6	41.37
Vitamin K, mg/kg	0.66	0.4
Kcal/g (DMB)	4.62	4.78
Kcal/g (as fed)	1.74	2.96
Ash, %	12.42	5.48

Note

“Days” is duration, with captive day 0 being their admittance to the PDRC (Athens, GA), for which nestlings consumed a specific diet phase. All nestlings received the same diet (natural) until they were in captivity for 113 days, after which the designated anthropogenic pen (*n* = 10) began receiving white bread and reduced seafood content. The day the new diet was given is considered to be experimental day 0. Dry matter is defined as the nonmoisture portion of a feed ingredient or diet. The sum of moisture and dry matter content of a feed on a percent of total will always equal 100. Dry matter contains the essential nutrients within a given feed ingredient or forage.

### 2.3 Collection of biological samples

For the first few weeks of maintenance, nestlings were handled every day to monitor their growth through obtaining a body mass and standard avian morphometric measurements (tarsus length, tarsus width, and culmen length), and to monitor for abnormalities (such as pododermatitis) or delayed growth. After 6.5 weeks in captivity, nestlings were transitioned to handling every other day and by 11 captive weeks were only handled weekly to obtain body weights, morphometric measurements or on an as‐need basis. Blood was collected (maximum of 1 ml) from the birds at 15 weeks in captivity for a pre‐diet bactericidal assay and measurement of plasma corticosterone.

**FIGURE 1:**
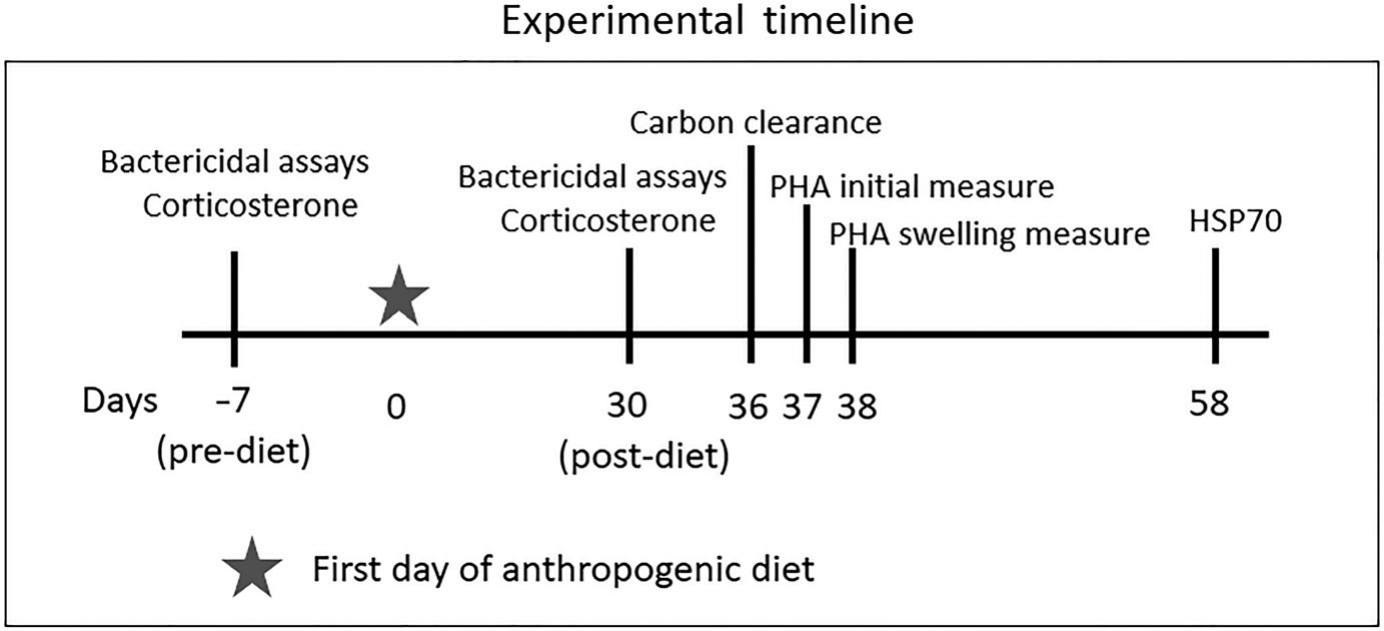
Timeline of white ibis nestling sample collection and the relevant assays samples were used for. Experimental Day 0 represents the day white bread was introduced into the diet, and seafood content was significantly reduced for 10 of the nestlings. In addition to these measures, body condition was also measured on experimental days −7 and 30 (before and after the experimental diets were implemented). Carbon clearance was the measure of phagocytic activity in blood samples collected from the nestlings. PHA, phytohemagglutinin

To determine the effects of the experimental diet on concentrations of corticosterone and HSP‐70, as well as measures of immunity, we collected blood 30 days after the start of the new diet for another bactericidal assay and corticosterone measurement, after 37 days of the diet for an in vitro phagocytosis assay called a carbon clearance assay, and after 58 days of the diet for measurement of HSP70 in circulation (see Figure 1 for a visual timeline). Blood was collected from either the jugular or the medial metatarsal vein using a 25‐gauge needle (Becton, Dickinson and Company). No more than 1% of the bird's body weight in blood volume was collected, and birds were never bled more than once in a 7‐day period. After collection, blood was transferred into 3 ml heparinized (for bactericidal assays and measurement of corticosterone and HSPs) or EDTA (for carbon clearance) vacutainer tubes (Becton, Dickinson and Company) and immediately placed on ice. When collected for use in bactericidal assays and corticosterone analysis, blood was transferred on ice back to the laboratory and centrifuged at 3,500 *g* for 10 min to obtain plasma. Approximately 80 μl of plasma was aliquoted into separate 2 ml cryovials (Corning), for either bactericidal or corticosterone analysis. Cryovials were then immediately placed in −80°C for storage until use in respective assays. The red blood cells remaining after centrifugation were transferred to a separate cryovial, stored −80°C for storage until used for heat shock protein analyses. The in vitro carbon clearance assay required 1 ml of whole blood, which was collected into EDTA‐lined tubes and then immediately transferred on ice to the laboratory for analysis. All animal procedures were reviewed and approved by the University of Georgia's Institutional Animal Care and Use Committee (AUP#A2016 09‐012).

### 2.4 Innate immunity—bactericidal capacity

We used bactericidal assays to measure in vitro bactericidal capacity of plasma using an assay following Matson et al. (2006) with minor modifications. Quantification of bactericidal capacity was performed with *Escherichia coli* from a working culture following manufacturer's instructions (*E. coli* ATCC 8739 pellets, Microbiologics) and *Salmonella paratyphi B* cultured from samples collected in ibis feces in the field. Aliquots (500 µl) of *E. coli* and *S. paratyphi* were made from dilutions of each bacteria with a mixture of 80% culture broth (Sigma‐Aldrich Inc) and 20% glycerol (VWR International) at a dilution which resulted in ~600 CFUs/10 µl when plated and incubated at 37°C. Aliquots were stored in 1.7 ml microcentrifuge tubes (Corning) in −80°C until use for these assays; ibis plasma was diluted with CO_2_‐independent media (Life Technologies) enriched with 4 mM L‐glutamine (VWR International). For use in *E. coli* assays, plasma dilutions were optimized to achieve an average of ~30%–80% killing using an initial pooled plasma sample resulting in a 1:15 (10 µl plasma + 140 µl media mixture) dilution of plasma. For *S. paratyphi* assays, we used a 1:2 (60 µl plasma + 60 µl media mixture) plasma dilution. Then, 120 µl of each plasma dilution was then separately combined with 40 µl of each bacterial aliquot (from premade aliquots) and left to incubate at room temperature for 30 min. Next, 40 µl of each bacterial aliquot was separately added to 120 µl of CO2‐independent media enriched with 4 mM L‐glutamine (no plasma), as positive controls with ~200 CFUs/50 µl, which were also incubated for 30 min at room temperature. Finally, 50 µl of all plasma‐bacteria combinations and controls was plated onto tryptic soy agar plates (VRW International) in duplicate and spread using a sterilized spreading wand. Plates were incubated at 37°C for 16 hr, at which time plates were removed to count the number of CFUs per plate. The average of the two plates per sample, including the controls, was taken; the bactericidal capacity (proportion of colonies that were killed relative to the number that grew on the control plate) for each sample was then calculated as.

$$\frac{\text{mean CFU control}‐\text{mean CFU sample}}{\text{mean CFU control}}$$

### 2.5 Innate immunity—phagocytic activity

An in vitro carbon clearance assay was used to evaluate phagocytic activity of macrophages, a function of innate immunity, following Spinu, Spinu, and Degen (1999) with slight modifications. Upon sample collection, 150 μl of each whole blood sample was added to individually labeled tubes containing 3 ml 0.09% saline for use as a background. The remainder (850 μl) of each sample (1:144 dilution in saline) received 5.9 μl of India Ink (Speedball Art Products) supernatant that had been centrifuged at 3,000 *g* for 30 min. Each of these samples was then vortexed to mix adequately then divided into five equal aliquots of approximately 170 μl each into 1.7 ml microcentrifuge tubes. Each of the five tubes from each bird was incubated for 10, 20, 30, 45, and 60 min at 37°C. After incubation, 150 μl of each blood and India Ink aliquot were added to pre‐labeled (e.g., Bird: X, Incubation Time: X) glass tubes containing 3 ml 0.09% saline. Each of these samples was then centrifuged at 50 *g* for 4 min. The absorbance of the supernatant of each sample was read with a spectrophotometer at 535 nm (SpectraMax Plus 384 multiplate reader, Molecular Devices), using the background of each individual as a blank prior to reading the blood and India Ink supernatant. The absorbance of the sample decreased over time as the carbon was phagocytosed. To calculate the rate of this phagocytosis for each sample, optical density readings were converted to a log2 scale and phagocytic index (change in optical density/min) was taken as the negative of the slope of the regression of optical density (log2) on time (min) for each bird.

### 2.6 Adaptive immunity—phytohemagglutinin (PHA) skin test

A phytohemagglutinin (PHA) skin swelling assay was used to evaluate T‐cell‐mediated immunity using a modification of the protocol described in to Smits, Bortolotti, and Tella (1999). After the injection of PHA, T‐cell migration into tissue is part of a localized inflammatory response. At 37 days after the nestlings were assigned to their diet treatment groups, each bird was injected intradermally with 0.1 mg PHA (Sigma Chemical Co.) that was suspended in 0.1 ml phosphate‐buffered saline (PBS) into the toe web between the second and third digits. Toe web thickness was measured immediately prior and 24 hr postinjection using a pressure‐sensitive micrometer and zeroing the micrometer between each measurement (Mitutoyo America Corporation); each toe web was measured twice, zeroing the micrometer between all measurements. All measurements and injections occurred on the toe web of the right foot, barring two individuals with open wounds on the plantar surface of their right feet for which we used the left foot to avoid confounding effects of ongoing inflammation. Toe web swelling was calculated as the difference in the average of the two measurements post‐injection and pre‐injection. To minimize experimental error, one investigator performed all injections and one performed all measurements. Smits et al. (1999) showed that a sham injection of PBS into the opposite toe web as a control is unnecessary, so this step was eliminated to minimize the psychological impact of handling and the chances of an injection error.

### 2.7 Plasma corticosterone measurements

Baseline plasma corticosterone levels were measured using an enzyme‐linked immunosorbent assay (ELISA; Corticosterone ELISA Kit, Enzo Life Sciences) following Herring, Cook, Gawlik, and Call (2011) with slight modifications. Kit instructions for small volume protocol for serum/plasma were followed, resulting in a 1:40 dilution of each plasma sample. Kit assay procedures were followed. The plate reader was blanked against the Blank Wells, and optical density of each well was read at 405 nm (Victor3 Basic, Perkin Elmer). Corticosterone Standard wells ranged from 32 pg/ml to 20,000 pg/ml; standards were used to calculate a standard curve on which sample concentrations of corticosterone were calculated using the mean optical density of each sample. Romero and Reed (2005) showed that corticosterone concentrations in birds had begun to increase by and even prior to 3 min post‐capture, so we did two things to minimize any confounds related to the psychological impacts of handling. First, we turned out all lights in the enclosures prior to sampling to calm the birds and reduce their ability to see us. We then quietly captured each bird and took a blood sample within a maximum of 3 min without disturbing the other birds (which is as quickly as we could collect the blood samples). In preliminary analyses, we found that corticosterone concentrations do not change in relation to amount of time since turning off the lights or time from capture to blood sampling within 3 min of capture (*p* > .30 in both cases).

### 2.8 Heat shock protein 70 (HSP70)

Concentrations of heat shock protein 70 (HSP70) in the blood were assessed as a secondary measure of physiological challenge following Herring et al. (2011). Heat shock protein 70 concentrations were measured at 23.5 weeks into captivity, 58 days after the nestlings were assigned to their diet treatment groups. Red blood cells were washed three times using phosphate‐buffered saline, centrifuged, and the supernatant was removed after the final wash. Red blood cell supernatant was then mixed with 1× extraction reagent and a protease inhibitor cocktail (Sigma), vortexed for 5 min, and then sonicated for 1 min. Samples were again centrifuged (15 min, 2,500 *g*), and the supernatant removed. We measured HSP70 in the supernatant using an ELISA kit (Enzo Life Sciences) specific to this protein. All samples were run in duplicate, and means of duplicates were used in all analyses.

### 2.9 Statistical analysis

To improve normality (Shapiro–Wilk's test) and homogeneity of variance (tested using Bartlett's test), we arcsine transformed all bactericidal and carbon clearance values. No other data required transformations. To test the influences of diet and sex of birds on measurements that were repeated before and after the initiation of dietary treatment (including body condition, bacterial killing ability against *E. coli* and *Salmonella*, and corticosterone concentrations), we used a general linear mixed effects model with diet, sex, time, and the interactions between diet and sex, diet and time, and sex and time as fixed effects. We used general linear models to test for effects of diet and on the swelling response to PHA, the phagocytic response to carbon particles, and the concentrations of HSP70 in circulation, using diet, sex, and the interaction of diet by sex as fixed effects. The lack of fit test was not significant for either model (*p* > .05). All statistical analyses were performed using JMP s. 14.1 (SAS Institute Inc., 1989–2019).

## 3 RESULTS

### 3.1 Body condition

The body condition of all nestlings increased significantly during the time the experimental diet was provided. There was also an effect of sex; males had significantly higher body condition indices than females. There was not a significant interaction of sex and time, indicating that body condition changed over the course of the experiment in a similar manner. There was no effect of diet, and no significant interaction between diet and time, indicating that diet did not influence the change in body condition over the course of the experiment (Table 3).

**TABLE 3:** Summary statistics for two linear mixed effects models testing the influences of diet and sex on (1) the change in body condition, and (2) plasma corticosterone concentrations before and after the experimental diet was started. Corticosterone values were log transformed for analysis. Diets included a “natural” diet that mimics what white ibis eat in the wild and an “anthropogenic” diet, which is higher in carbohydrates and lower in protein. Body condition was significantly higher in males at all sampling points (Males: Mean = 10.83, Females: Mean = 9.57), and increased between the pre‐ and post‐diet body condition measures (pre‐diet mean: 10.02, post diet mean: 10.77). Plasma corticosterone concentrations also increased significantly between pre and post‐diet measures (pre‐diet: 4.20 ng/ml, post‐diet: 11.92 ng/ml)

Parameter (*df*)	Body condition					Plasma corticosterone				
	*F*	*p*	Effect size	Lower 95%	Upper 95%	*F*	*p*	Effect size	Lower 95%	Upper 95%
Diet	0.16	.69	0.003	−0.06	0.07	0.01	.94	0.14	−3.46	3.74
Time	18.60	<.01	0.002	−0.02	0.02	7.89	.01	−4.86	−8.51	−1.21
Sex	6.90	<.02	0.01	−0.05	0.08	0.01	.93	0.15	−3.45	−3.75
Diet × time	2.52	.13	0.03	0.01	0.04	0.01	.95	0.10	−3.55	3.75
Diet × sex	0.33	.57	−0.003	−0.07	0.06	0.86	.37	1.57	−2.02	5.17
Sex × time	0.13	.72	−0.002	−0.02	0.02	0.42	.52	1.12	−2.53	4.78

### 3.2 Corticosterone and heat shock protein 70

**FIGURE 2:**
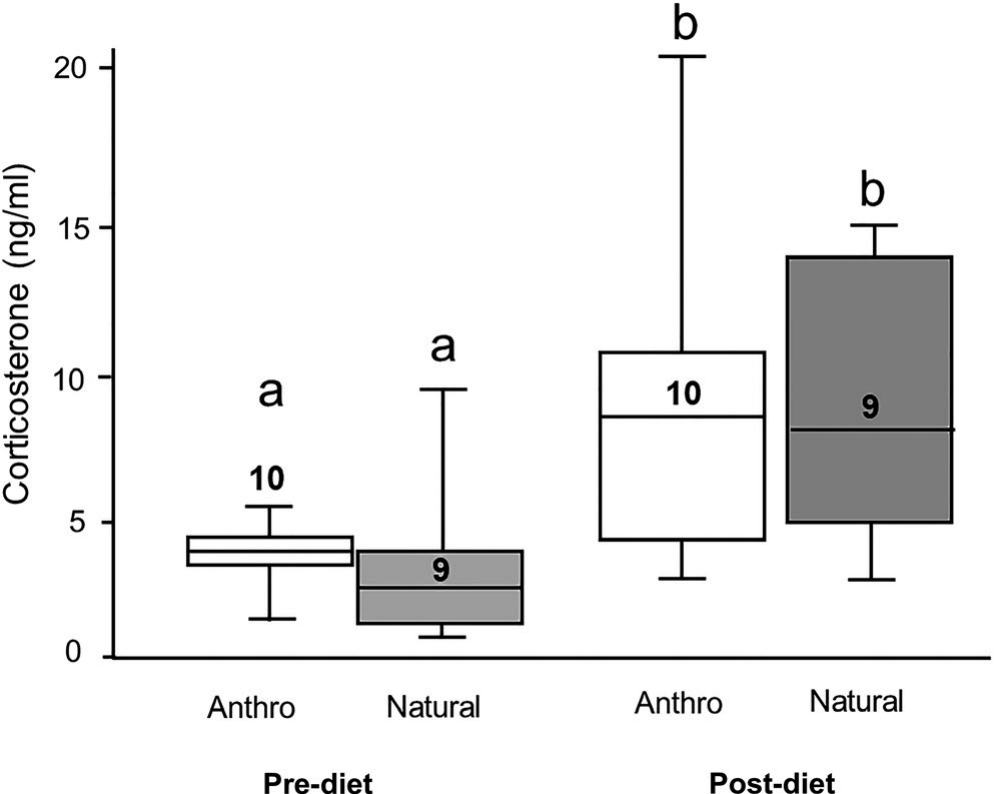
Relationship between experimental diet and concentrations of corticosterone (means ± *SE*) in plasma before and 30 days after the experimental diet was implemented. All 20 nestlings were sampled for corticosterone. One postdiet sample was excluded because it took more than 3 min to collect that sample. Otherwise, all were collected within 3 min of capture. There were statistical outliers in both the pre‐ and postdiet groups (prediet: 1 natural diet bird = 14.88 ng/ml, postdiet: 1 natural diet bird = 53.11 ng/ml). We left these in the analyses because we had no good reason to remove them, and their presence/absence did not change the overall result, but we removed them for the purposes of depicting the data in a way they were visible. Significant differences are indicated by different letters over boxes, and numbers in (or just above) boxes indicate sample sizes. For these box plots, the boxes show the 25th and 75th percentile values, the line within shows the median value, and the whiskers show the minimum and maximum of the dataset

**FIGURE 3:**
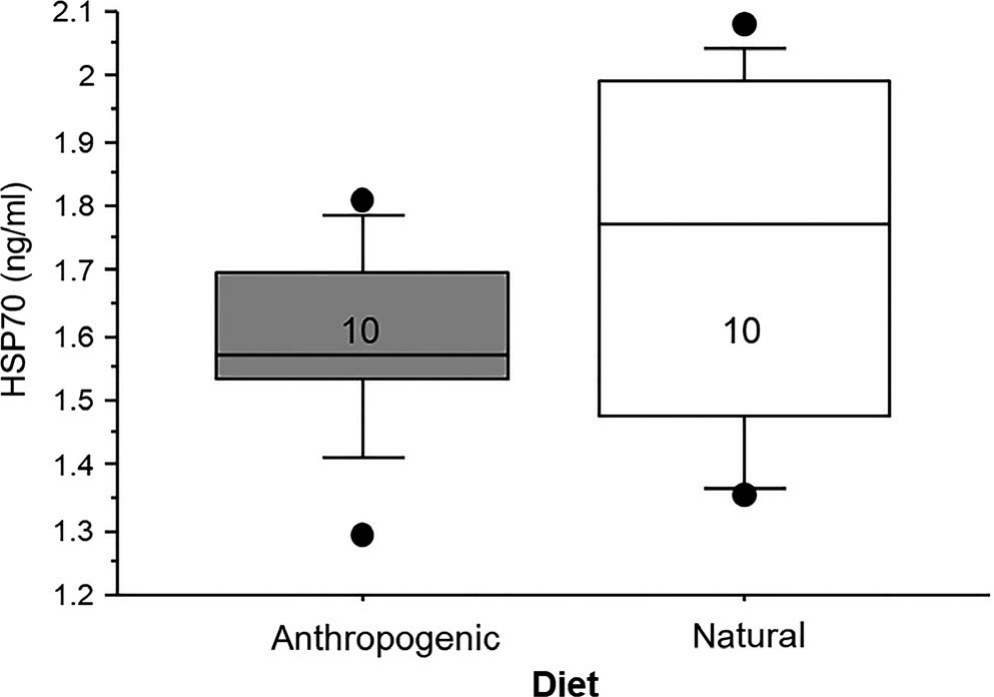
Box plots showing the relationship between experimental diet and concentrations of heat shock protein 70 (HSP70, ng/ml) in blood. There was no significant influence of diet on this measure. Numbers in the boxes indicate sample sizes. For these box plots, the boxes show the 25th and 75th percentile values, the line within shows the median value, and the whiskers show the minimum and maximum of the dataset, with additional data points showing potential outliers in the dataset

Plasma corticosterone concentrations were similar between males and females, but increased significantly by 30 days after the initiation of the experimental diets. There was no effect of diet on concentrations of corticosterone in plasma, and there was no interaction between diet and time, indicating that the changes in corticosterone concentrations were not an effect of the anthropogenic diet (Table 3, Figure 2). There was also no influence of diet or sex on concentrations of HSP70 in circulation after 58 days on the experimental diets (Table 4, Figure 3).

**TABLE 4:** Summary statistics for a general linear model testing the effects of diet on the concentration of heat shock protein 70 (HSP70), which is an indicator of physiological challenge and cellular damage

	Heat shock protein 70				
	*F*	*p*	Effect size	Lower 95%	Upper 95%
Diet	−1.61	.13	−0.07	−0.17	0.02
Time	1.52	.15	0.06	−0.03	0.16
Sex	−1.80	.09	−0.08	−0.17	0.01

### 3.3 Immunological measures

**FIGURE 4:**
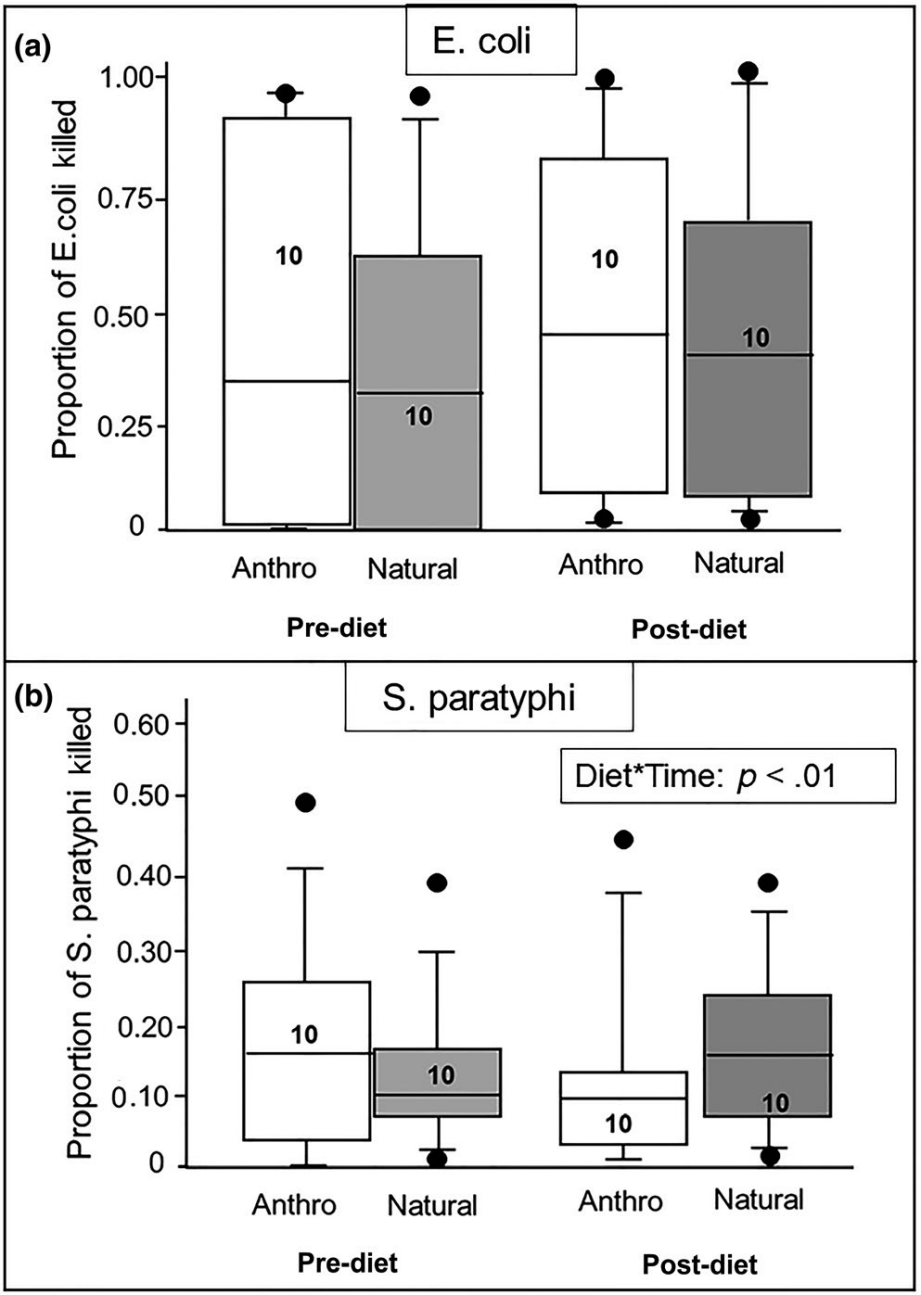
Relationship between diet and the bactericidal capacity (mean proportion of killed bacteria relative to colony growth on a control plate ± *SE*) against (a) *Escherichia coli* and (b) *Salmonella* *paratyphi*. Measurements were made before and 30 days after implementation of the experimental diet. There was no effect of sex, diet, or time on the ability of ibis blood to kill either bacterial species. There was a significant diet × time interaction on the bactericidal capacity against *S. paratyphi*. While birds in the natural diet group tended to get better at killing *S. paratyphi*, birds receiving anthropogenic tended to get worse, indicating that the implementation of the experimental diet influenced the ability to kill *S. paratyphi.* The bacterial killing capacity for both bacterial species was arcsine transformed for analyses, but raw data are presented here. Numbers in the bars indicate sample sizes. For these box plots, the boxes show the 25th and 75th percentile values, the line within shows the median value, and the whiskers show the minimum and maximum of the dataset, with additional data points showing potential outliers in the dataset

**FIGURE 5:**
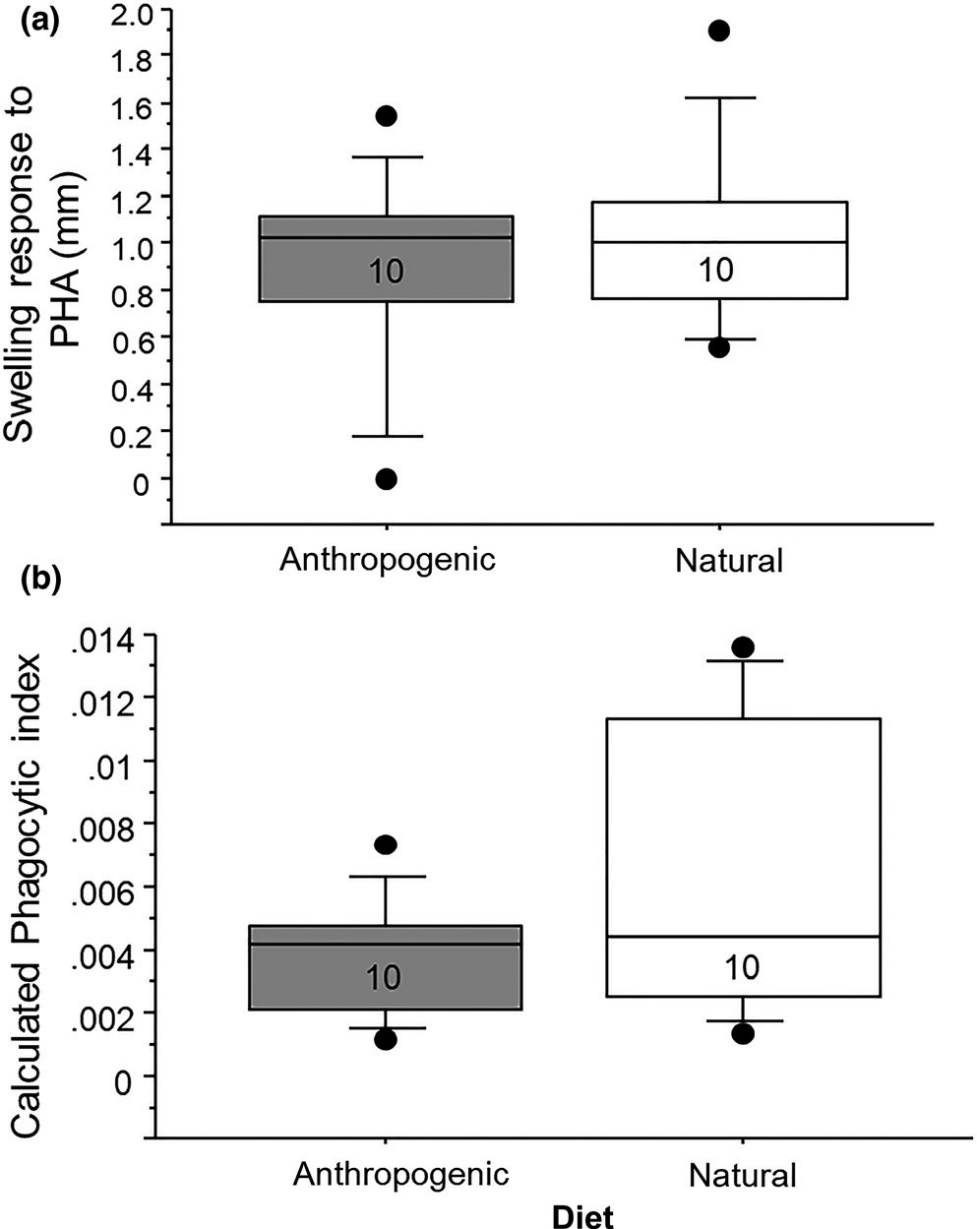
Relationships between dietary treatment and (a) swelling response (mm) against phytohemagglutinin (PHA, measured 38 days after initiation of experimental diets) and (b) the ability of the blood to clear carbon particles via phagocytosis (presented as calculated phagocytic index, the change in optical density readings over time). There was no effect of diet on either of these measures. Numbers in the boxes indicate sample sizes. For these box plots, the boxes show the 25th and 75th percentile values, the line within shows the median value, and the whiskers show the minimum and maximum of the dataset, with additional data points showing potential outliers in the dataset

Bactericidal capacity against both *E. coli* and *S. paratyphi* was similar between the two sexes. While the ability to kill *E. coli* was marginally better in all birds after the experimental diets were given, this different was not significant (*F*_1,17_ = 4.10, *p* = .06) and there was no significant effect of diet, nor was there a significant interaction effect between diet and time (Table 5, Figure 4). In comparison, there was a significant interaction between diet and time on bactericidal killing capacity against *S. paratyphi*; A *t* test comparing the change in bactericidal capacity between dietary treatment groups indicated that bactericidal capacity of control birds increased after the experimental diets started, but decreased in birds fed the anthropogenic diet (*t*_1,18_ = −3.07, *p* < .01). (Table 5, Figure 2). Neither the PHA‐induced skin swelling nor phagocytic activity (as indicated by the carbon clearance test) differed between the sexes or between the dietary treatment groups (Table 6, Figure 5a,b).

**TABLE 5:** Summary statistics for two linear mixed effects models testing the influences of diet and sex on bactericidal capacity againsted (1) *Escherichia coli* and (2) *Salmonella paratyphi* before and after the experimental diet was started. Bactericidal percentages were arcsin transformed for analysis. Diets included a “natural” diet that mimics what white ibis eat in the wild and an “anthropogenic” diet, which is higher in carbohydrates and lower in protein. There was a significant diet x time interaction effect on bactericidal capacity against *S. paratyphi*

	*Escherichia coli* killing					*Salmonella* * paratyphi* killing				
	*F*	*p*	ES	Low 95%	Upper 95%	*F*	*p*	ES	Low 95%	Upper 95%
Diet	0.04	.85	0.04	−0.22	0.27	0.01	.92	0.003	0.08	0.22
Time	4.10	.06	−0.07	0.15	0.00	0.17	.78	−0.002	−0.02	0.02
Sex	0.29	.59	0.06	−0.18	0.30	0.08	.69	−0.012	−0.06	0.07
Diet × time	0.01	.95	−0.001	−0.07	0.07	9.14	<.01	0.03	0.01	0.04
Diet × sex	0.02	.88	−0.02	0.27	0.22	0.01	.92	−0.003	−0.07	0.07
Sex × time	0.06	.80	0.009	0.06	0.08	0.03	.85	−0.002	−0.02	0.02

**TABLE 6:** Summary statistics from two general linear models testing the effects of diet on the swelling response to phytohemagglutinin and the phagoctyic ability of blood, measured using a carbon clearance assay

	PHA‐induced swelling					Carbon clearance (Phagocytosis)				
	*F*	*p*	ES	Low 95%	Upper 95%	*F*	*p*	ES	Low 95%	Upper 95%
Diet	−0.62	.54	−0.06	−0.27	0.15	−1.53	.15	−0.001	−0.003	0.004
Sex	0.07	.94	0.007	−0.20	0.21	−0.56	.58	0	−0.002	0.001
Diet × sex	0.57	.57	0.06	−0.15	0.26	1.14	.27	0.001	−0.001	0.003

## 4 DISCUSSION

In this study, we aimed to understand how an anthropogenic diet may affect several physiological indicators of physiological challenge and immune function in a bird species that has only recently begun feeding from anthropogenic sources on a routine basis. Previous studies have shown that energy‐rich diets improve overall health via a reduction in glucocorticoid concentrations in circulation and an improvement in immunity (Strandin, Babayan, & Forbes, 2018; Wilcoxen et al., 2015). Because anthropogenic diets that ibises consume contain a different nutrient profile than they would find in more natural food sources, we predicted that feeding ibises anthropogenic food sources would exert significant effects on immune responses and indicators of physiological challenge. We found that consuming our experimental anthropogenic diet did not appear to trigger increases in these indicators of physiological challenge (corticosterone and HSP‐70), but decreased the development of the capacity to kill *S. paratyphi*.

Concentrations of corticosterone in the plasma of nestlings increased significantly before and after the experimental diets were started, though the birds in the two dietary treatment groups showed similar increases in this measure. Additionally, our second indicator of physiological challenge, HSP70, was similar between the two treatment groups after the birds were on the experimental diet for almost two full months. If the provided anthropogenic diet served as a true physiological challenge to the animal, we would have expected to see elevated levels of corticosterone and HSP70 in blood. It is possible that the psychological impacts of captivity may have cloaked any effects of diet that would be seen in wild ibises; however, concentrations of corticosterone in these nestlings were, for the most part, similar to baseline levels observed in wild ibises (Curry, 2017). Two birds exhibited quite high concentrations of corticosterone, but they were split between the two treatment groups, and results did not change if these birds were removed from the analyses. Thus, we believe that this result indicates that the anthropogenic diet did not provoke changes in these two indicators of physiological challenge in birds, nor did it appear to relieve unnatural elevations of these mediators. While many describe corticosterone and HSP‐70 as a “stress indicators,” they are actually a functional mediator of metabolic processes and protectors against cellular damage, respectively. As a result, they can both be active even when an animal is not experiencing what some refer to as “stress” (MacDougall‐Shackleton, Bonier, Romero, & Moore, 2019). So, while an anthropogenic diet may not trigger a large increase in circulating concentrations of either corticosterone or HSP‐70, these mediators may still mediate metabolic effects that can influence different aspects of the immune system. This remains to be tested.

Bactericidal capacity, on the other hand, was influenced by the dietary treatments (Table 5). Ibises on the natural diet showed on average a 4% increase in their ability to kill *S. paratyphi* over time, while those on the anthropogenic diet showed a 5% decrease in their ability to kill the same bacterial species. Statistical tests indicated that the change in bactericidal ability against this bacterial species between pre‐ and post‐dietary manipulation differed significantly between the dietary treatment groups. This indicates that consuming an anthropogenic diet may prohibit the full development of the mechanism necessary for killing *S. paratyphi*.

There was no effect of diet on any other measure of immune function, including the swelling response to PHA, phagocytic response indicated by the ability of the blood to clear carbon, and even the bactericidal capacity against a different bacterial species, *E. coli*. It is not surprising that one measure of immunity was affected by diet while others were not. The immune system is extremely complex, and we would not necessarily expect the different aspects of the immune system to respond in similar ways to physiological challenges. In fact, we saw a *negative* relationship between bactericidal capacity (against both *E. coli* and *S. paratyphi B*) and the PHA‐induced swelling responses. Bactericidal responses are part of the innate immune system and involve the activity of complement (Merchant, Roche, Elsey, & Prudhomme, 2003). The swelling response to PHA, on the other hand, involves T‐lymphocytes, which are part of the adaptive immune response (Tella, Lemus, Carrete, & Blanco, 2008). Immune system components often undergo energetic trade‐offs, particularly between adaptive and innate immunity (Bourgeon, Kauffmann, Geiger, Raclot, & Robin, 2010), so this may be why we saw the inverse relationship between the two immunological measures. But why the different patterns between the two bactericidal measures? The process of bacterial killing via complement is complex; complement can mark the bacteria for destruction by phagocytes or bind itself to the bacteria and activate proteases that kill the bacteria (Blom, Hallström, & Riesbeck, 2009). As a result, the mechanisms by which *E. coli* and *S. paratyphi* could differ, and the availability of cell types and/or functional components needed to carry out the bactericidal activity for each bacterial type could be differentially influenced by the nutritional content of the diet. It is interesting that only the response against the more ecologically relevant bacterial species was affected, as *S. paratyphi* is a pathogenic strain of bacteria that these ibises are exposed to in the wild. Additionally, ibises found in urban parks have higher loads of Salmonella (Hernandez et al., 2016). More work needs to be done to explore the precise mechanisms by which bactericidal activity occurs in plasma, and how those mechanisms relate to the consumption of different micro‐ and macronutrients.

While we saw an effect of diet on the ability to kill *S. paratyphi*, it is possible that we would have seen influences of diet on more of the measured physiological parameters if we had supplied the experimental diet for longer, provided a more extreme dietary manipulation, and/or sampled a larger quantity of birds. Captive diet manipulation experiments range in length, with some studies subjecting experimental animals to a certain diet for as little as 15 days (Acquarone, Cucco, Cauli, & Malacarne, 2002) and some up to as many as 59 days or beyond after the start of experimental feeding (Smith, Råberg, Ohlsson, Granbom, & Hasselquist, 2007). Ibis in the wild experience fluctuations in resource availability due to fluctuating water levels, so this species may not be as sensitive to short‐term changes in diet as one might expect. Still, this also argues that our study design was, in fact, appropriate, because wild white ibises would not likely experience a diet change that lasted for longer than 30 consecutive days.

Our goal in this study was to limit overall protein intake, but we needed to provide a nutritionally balanced diet (20% seafood, 20% flamingo pellets, and 20% eggs) in addition to the white bread (40%) because nestling health was a priority. Further, we also provided the flamingo pellets to the control diet as a base, and these pellets do contain some carbohydrates that wild ibises may not normally take in. These are limitations to the study that were unavoidable because we wanted to ensure that the ibises survived in captivity. In addition, it is known that while in urban habitats, ibis continue to forage on terrestrial invertebrates, so the effects of diet may be dependent on exact proportions of anthropogenic food to natural prey. In the future, additional studies are needed to test the effects of each dietary component by dramatically decreasing or eliminating those components in experimental diets.

Overall, the experimental anthropogenic diet did not appear to trigger elevations in corticosterone or HSP‐70 in white ibises, and only impacted one measure of immunity to a relatively small degree. We initially hypothesized that the anthropogenic diet would increase indicators of physiological challenge and decrease measures of immune function. We did see that the development of the ability of the ibises to kill *S. paratyphi*, the more ecologically relevant of the two bacterial species, appeared to be hindered by consumption of the anthropogenic diet, but this did not result in statistical differences in this measure of immunity between the two treatment groups after the diet was given. This indicates that the anthropogenic diet we gave did not truly represent a large physiological challenge to the birds, as we had initially predicted. In future studies, it is important to measure additional immunological parameters that we did not measure in the current study. For example, antibody responses are particularly important during immunological challenge and should be measured in relation to anthropogenic feeding. In addition, further work needs to be done to assess whether a diet that is less nutritionally complete may more potently influence indicators of physiological challenge and immunity in this species, and additional tests need to be done to assess the impacts of other factors related to foraging in an urban environment, such as anthropogenic noise and direct exposure to humans.

## CONFLICT OF INTEREST

The authors have no competing interests.

## AUTHOR CONTRIBUTIONS

**Caroline R. Cummings:** Formal analysis (supporting); investigation (lead); methodology (lead); writing – original draft (lead); writing – review and editing (supporting). **Sonia M. Hernandez:** Conceptualization (lead); funding acquisition (lead); investigation (lead); methodology (lead); project administration (lead); resources (lead); supervision (equal); writing – review and editing (supporting). **Maureen Murray:** Conceptualization (equal); data curation (equal); formal analysis (equal); investigation (equal); methodology (equal); project administration (equal); supervision (equal); writing – review and editing (supporting). **Taylor Ellison:** Methodology (supporting); project administration (supporting). **Henry C. Adams:** Conceptualization (supporting); investigation (supporting); methodology (supporting); project administration (supporting). **Robert E. Cooper:** Conceptualization (supporting); methodology (supporting); project administration (supporting). **Shannon Curry:** Conceptualization (equal); data curation (equal); formal analysis (equal); investigation (equal); methodology (equal); project administration (equal); validation (lead). **Kristen J. Navara:** Conceptualization (lead); formal analysis (lead); funding acquisition (equal); investigation (equal); methodology (equal); project administration (equal); validation (equal); writing – original draft (equal); writing – review and editing (equal).

## Data Availability

Data described herein have been archived the Dryad public repository, https://doi.org/10.5061/dryad.ns1rn8pq0.
